# Three-Dimension-Printed Custom-Made Prosthetic Reconstructions in Bone Tumors: A Single Center Experience

**DOI:** 10.3390/curroncol29070361

**Published:** 2022-06-28

**Authors:** Raffaele Vitiello, Maria Rosaria Matrangolo, Alessandro El Motassime, Andrea Perna, Luigi Cianni, Giulio Maccauro, Antonio Ziranu

**Affiliations:** 1 Orthopedics & Traumatology Unit, Fondazione Policlinico Universitario Agostino Gemelli IRCSS, 00168 Roma, Italy; lele.vitiello@gmail.com (R.V.); mariarosaria.matrangolo01@icatt.it (M.R.M.); andrea.perna01@icatt.it (A.P.); luigi_cianni@libero.it (L.C.); giulio.maccauro@policlinicogemelli.it (G.M.); antonio.ziranu@policlinicogemelli.it (A.Z.); 2 Orthopedics and Traumatology, Università Cattolica Del Sacro Cuore, 00168 Roma, Italy

**Keywords:** bone tumors, computer-aided design, 3D-printed prosthesis, reconstruction surgery, custom-made prosthesis, large bone defects

## Abstract

Bone can be affected by different neoplastic conditions. Limb salvage surgery has become the preferred treatment strategy for most malignant tumors of the extremities. Advanced 3D printing technology has transformed the conventional view of oncological surgery. These types of implants are produced by electron beam melting (EBM) technology by sintering titanium powder in a scaffold shape designed following a project designed from HRCT and MRI. The aim of our study was to evaluate the outcomes and the mid-term follow-up of a population treated with 3D-printed custom-made prosthesis implantation in major oncological bone resection or after failure of primary implants. The primary outcome was the general patient satisfaction one year after surgery. The secondary outcomes were: mortality rate, treatment related complication rate, functional and clinical outcomes (KPS, ADL and IADL). Eight patients were included, five females and two males, with a mean age of 50.3 (±23.72) years at the surgery. The enrolled patients reported a mean satisfaction rate after surgery of 7.38 (±2) where 10 was the maximum value. There were no changes between pre- and postoperative mean KPS (81.43 +/−10.69). Mean preoperative ADL and IADL score was in both cases 4.86 (±1.07), while postoperative was 5 (±0.82), with a delta of 0.13 (*p* > 0.05). Custom-made prosthesis permits reconstructing bone defects caused by large tumor resection, especially in anatomically complex areas, restoring articular function.

## 1. Introduction

Bone can be affected by different neoplastic conditions; these include primary bone tumors, lymph-hematologic neoplasms and metastatic disease from distant primary sites, especially from the breast and prostate [1].

Primary bone tumors are uncommon, accounting for less than 0.2% of all malignancies. Each tumor subtype has a different pattern of occurrence, but none of them have more than 0.3 new cases per 100,000 people per year [1]. Osteosarcoma, Ewing’s sarcoma and chondrosarcoma are the most common malignant primary bone tumors, which account for 70% of all such malignancies. The first two are relatively frequent in the second decade of life, while OS is more common in older age [2,3].

On the other hand, bone is a particularly common site of metastases and affects a large number of individuals with advanced cancer [3]. The manifestation of skeletal metastases is a significant event that negatively impacts the prognosis of those patients [4]. The most common sites of metastases are the spine (87%), pelvis (63%), skull (35%) and ribs (77%) as well as the proximal humeri and femora (53%); the distal appendicular skeleton is a quite uncommon site (1%) [3,5].

The diagnosis and management of such neoplasms require a multidisciplinary approach that includes orthopedic surgeons, radiologists, pathologists, and oncologists.

For primary or single/oligo-metastatic malignant bone tumors, wide excision is considered the principal treatment of choice [6].

In the past, bone sarcomas were usually treated with amputation of the affected limb. Nowadays, owing to early diagnosis, advances in chemotherapy and innovation in surgical techniques and the medical device industry, limb salvage surgery has become the preferred treatment strategy for most of primary and secondary malignant tumors of the extremities [7,8]. Reconstruction options after bone resection include bone graft (such as autograft, allograft), bone substitutes and modular prostheses, which is the most used technique [9,10].

Although reconstructions with autograft and allograft seem to have a lower incidence of long-term complications and acceptable joint function, they are related to several early complications. Furthermore, while prosthetic replacements provide a satisfactory and relatively early functional restoration, long-term failure rates, particularly in younger patients, have been reported to be significant [11].

Advanced 3D printing technology has recently transformed the conventional view of oncological surgery, bringing an accurate tumor removal and a patient-tailored reconstruction [8].

These types of implants are custom-made for the defect; they are produced by electron beam melting (EBM) technology, which allows the porous structure of the implant to integrate with the host bone [12].

This technology is now rapidly gaining relevance even in other fields such as maxilla-facial surgery for mandible or orbital reconstruction, or in otorhinolaryngology for ossicular chain reconstruction, and the aerospace sector.

The 3D-printed customized prosthesis are tailored to the patient’s anatomy; those are particularly useful in specific skeletal segments, where the reconstruction of large bone defects, caused by tumor resection, is extremely complex, giving a full restoration of articular function and reducing the incidence of complications [13].

The advantages of using such technology include the opportunity to increase skeletal reconstruction accuracy by making implants with complex shapes, reducing the risk of mismatch between the prostheses and host bone; allowing host bone to grow inside the implant, due to the superficial porosity of the prostheses, that provides a more stable reconstruction and full function of the body segment; mechanical strength to support the body weight, owing to the use of titanium to create these prostheses; the availability in most institutions [12,14,15,16].

The aim of our study was to evaluate the outcomes and the mid-term follow-up of a population treated with 3D-printed custom-made prosthesis implantation in the major oncological bone resection or after failure of primary implants.

## 2. Materials and Methods

### 2.1. Study Setting and Design

A retrospective observational study according to the PROCESS guidelines [17] was conducted on 14 patients treated with custom-made 3D prosthesis at our University Hospital between March 2016 and March 2021. As this is an approval from the Review Board of Orthopedic and Traumatology Institute there is no code. The approval date is the session of 22 June 2021. The study respects national ethical standards and the Declaration of Helsinki. Written informed consent for surgical and clinical data collection for scientific purposes were obtained from all patients at the admission and before surgery according to institutional protocol.

### 2.2. Inclusion and Exclusion Criteria

Inclusion criteria were: (I) pathological diagnosis of a primary malignant bone tumor or metastatic bone lesion; (II) extensive bone loss that excluded the use of standard prostheses (II) use of 3D printed custom-made prosthesis; (III) the consent of the patient to be included in the study.

Exclusion criteria were: (I) less than one year of follow up, (II) incomplete radiological and clinical data set.

### 2.3. Perioperative Management

**Figure 1:**
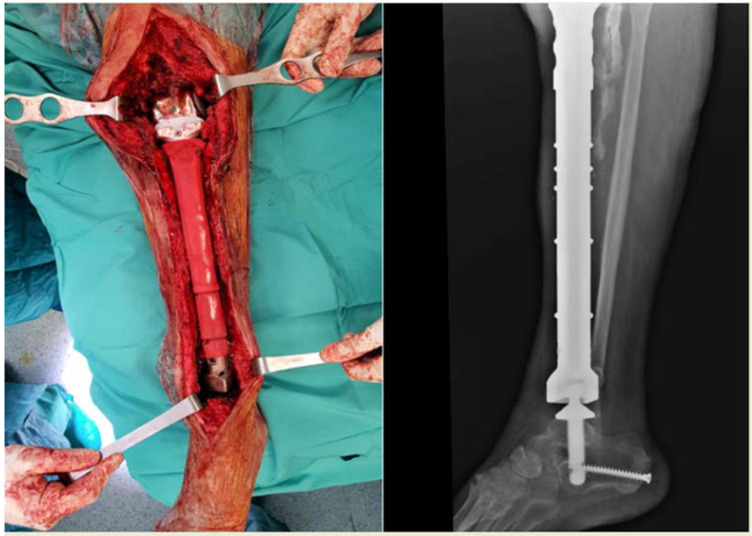
Patient affected by adamantinoma of the tibia underwent total excision and replacement with custom-made prostheses.

**Figure 2:**
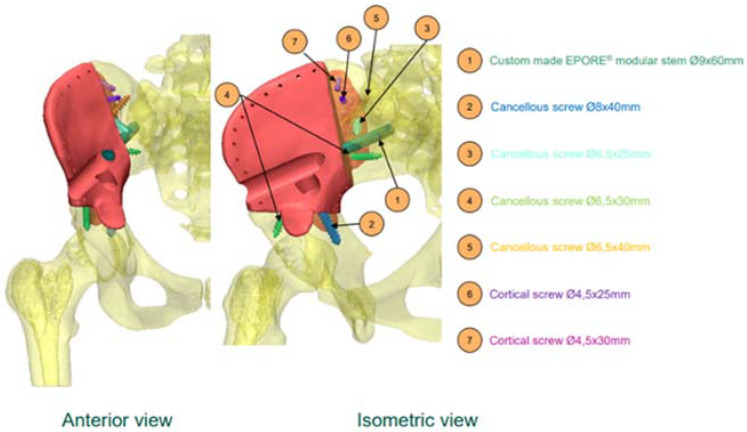
Pre-surgical planning.

**Figure 3:**
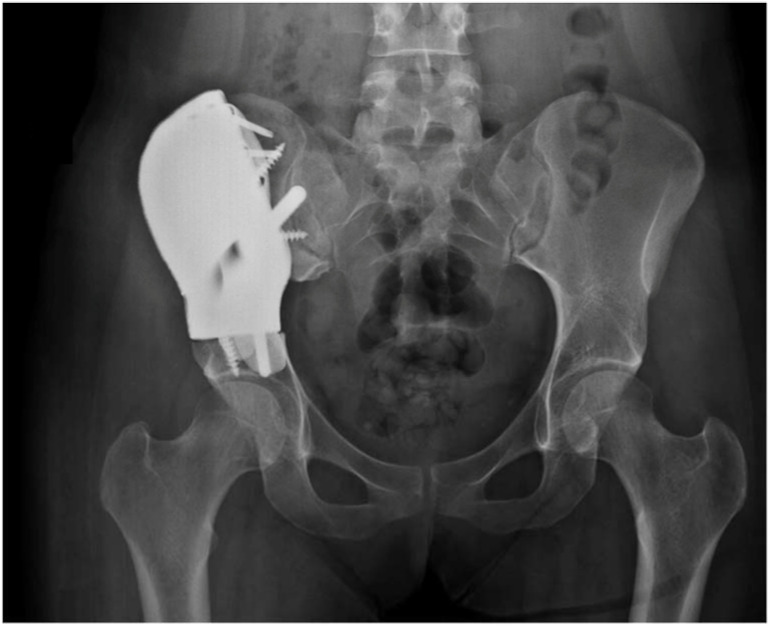
Patient affected by symptomatic fibrous dysplasia underwent total excision and replacement with custom-made prostheses.

All patients underwent preoperative radiography, high-resolution computed tomography (CT) (1 mm thin layer) and magnetic resonance imaging (MRI) of the affected segment. The images were sent to the manufacturing company, and a precise 3D virtual image and project was developed for each patient. The images, stored in DICOM (Digital Imaging and Communication in Medicine) format, were processed by a CAD (Computer Aided Design) software and, supported by an engineer’s input and surgeon’s input, the project and then the final product were realized. The prosthesis was printed using EBM technology, by sintering titanium powder (titanium alloy Ti6Al4V) layer after layer fully melted (50 μm each) at a speed of approximatively 4 mm/s having a stable temperature of around 700 °C during the whole process [18]. A porous structure very similar to bone tissue and its trabecular morphology is the result of this process. All prostheses were manufactured by the same company (Implantcast Ltd., Buxtehude, Germany) and all of them, in order to reduce infectious complications, were silver coated. To get it ready for surgery, at the end of the process the prosthesis was sterilized. Soft tissue reconstruction around the 3D printed customized prosthesis was obtained in all patients (excluding iliac bone surgery) using a Trevira Tube (Mutars^®^, Implantcast Corp., Buxtehude, Germany) made of polyethylene terephthalate (PET). For iliac bone reconstruction, the 3D prostheses allow the insertion of tendons and ligaments in specific sites of the implant. (Figure 1, Figure 2 and Figure 3).

All the procedures were performed by the same surgeon, an expert in oncologic orthopedic surgery (G.M.). Preoperative antibiotic prophylaxis, with intravenous Cefazolin 2 g, were administered to all patients, as per protocol in oncological patients [19,20].

A urinary catheter was preoperatively placed. After the surgical procedure, a drainage tube was placed in all patients. In all cases, the surgical drainage tube was removed 3 days after surgery, while the urinary catheter 1 day after patient mobilization.

Postoperatively, anti-thrombotic stockings and low molecular weight heparin prophylaxis were used, in order to avoid deep vein thrombosis. Progressive weight bearing was allowed in patients with pelvic and lower limb implants after 40 days from surgery. Immobilization with a Walker-brace after distal tibia reconstruction, with a Don-joy brace after knee reconstruction and with a stockinette-Gilchrist bandage after humeral reconstruction were performed. In patients with upper limb reconstruction, after 30 days rehabilitation was allowed.

### 2.4. Demographics, Comorbidity and Complications

Demographics and comorbidity data of all patients were investigated and summarized in Table 1. Furthermore, we evaluated pre- and post-operative laboratory data, such as preoperative and postoperative hemoglobin values, creatinine values, albumin values and Neutrophil to lymphocyte ratio (NLR) and platelet to lymphocyte ratio (PLR).

Complications were considered as intraoperative, early (within 6 postoperative months), and late (more than 6 months after surgery). We evaluated urinary tract infections, periprosthetic infections, aseptic loosening, wound infections, and tumor recurrence. Second-level exams, such as CT scans or MRI, were performed in case of wound dehiscence, local pain and signs of local inflammation. A superficial wound infection was defined by the absence at the CT or MRI images of periprosthetic fluid collection. All cases were discussed with an infectologist to define the therapeutic approach.

### 2.5. Clinical and Radiological Follow Up

Each patient was systematically clinically and radiological monitored at one, three, six and twelve months after surgery and then once a year. A survey was conducted to assess the quality of life of each patient before and after surgery. The Karnofsky performance status (KPS) is an assessment tool for functional impairment, determining the ability of oncologic patients to accomplish ordinary tasks. The KPS scores range from 0 to 100: the higher the score, the better the abilities of the patient to carry out daily activities [21]. Instrumental activities, daily living (IADL) and Activity of Daily Living (ADL) scales were also used. ADL refers to activities that are focused on taking self-care of themselves and their body, involving activities like toileting, dressing, bathing and eating. The score ranges from 0 to 6. IADL refers to activities, in the home and community, that support daily living and they need more elaborate interactions than ADL. Managing finances, housework, grocery shopping, making phone calls, and administering medications are examples of such activities. The score ranges from 0 (the patient is totally dependent) to 8 (independent) [22]. SF-12 scale has two summary measures: the Physical (PCS-12) and Mental (MCS-12) Component Summary scores, evaluating physical health and emotional well-being [23].

### 2.6. Satisfaction Assesment

During the 12-month follow-up visit, patients expressed their general satisfaction about receiving treatment through a 11-itemized satisfaction scale. Values ranging from 0 to 4 reflect a general dissatisfaction. Values ranging from 5 to 7 reflect an acceptable satisfaction after surgical treatment. Values ranging from 8 to 10 reflect an excellent satisfaction 1 year after surgery [24].

### 2.7. Outcomes

The primary outcome was the general patient satisfaction one year after surgery. The secondary outcomes were: mortality rate, treatment-related complication rate, functional and clinical outcomes (KPS, ADL and IADL).

### 2.8. Statistical Analysis

Dedicated SPPS statistical calculation software (SOSS Inc., Chicago, IL, USA) was employed. Data will be described using means and standard deviations for quantitative variables and numbers and percentages for qualitative variables. Only two decimal digits were reported, rounded up. Student’s *t*-test was used for statistical analysis of continuous variables.

## 3. Results

### 3.1. Patients Population

Eight patients were included in the study, five females and two males, with a mean age of 50.3 (±23.72) years at surgery. The mean BMI was 26.4 (±4.4) kg/m^2^.

The implant sites were as follows: 2 iliac bone, 2 distal tibiae, 1 proximal humerus, 1 distal femur, 1 hip and 1 total tibia (see Table 1 and Table 2). The diagnoses were different among the patients: osteosarcoma, adamantimoma, dedifferentiated sarcoma, chondrosarcoma, desmoplastic fibroma, osteofibrous dysplasia. Revision oncologic surgery was performed only in one patient after a breakage of femoral–tibial modular prosthesis. The principal features and demographic data of the involved patients were reported in Table 1.

### 3.2. Perioperative Data

Mean preoperative hemoglobin value was 13.24 (±1.77) g/dL, while on the first postoperative day it was 9.58 (±1.47) g/dL (*p* < 0.0032). Preoperative NLR and PLR values were respectively 2.00 (±0.51) and 98.9 (±44.9) (see Table 3). A tumor en-bloc resection with wide surgical margins was performed for each oncological patient enrolled. Urinary catheter was removed 6.6 (±2.1) days after surgery, while drainages were removed on the third postoperative day.

The mean length of hospitalization was 30.25 (±12.5) days and the mean follow-up was 21.17 (±9.4) months.

### 3.3. General Satisfaction and Clinical Outcomes

The enrolled patients reported a mean satisfaction rate after surgery of 7.38 (±2) where 10 was a maximum value. All patients except one reported an excellent satisfaction with received treatment. 

There were no changes between pre- and post-operative mean KPS (81.43 ± 10.69) (see Table 4). Mean preoperative ADL and IADL score was in both cases 4.86 (±1.07), while postoperative was 5 (±0.82), with a delta of 0.13 (*p* > 0.05). Preoperative M-SF-12 mean score was 11.71 (±0.49), while postoperative was 11.57 (±0.79) (*p* > 0.05); mean preoperative value of P-SF was 14.29 (±1.11) and changed postoperatively to 14.43 (±1.27) (*p* > 0.05).

### 3.4. Complications and Mortality

No intraoperative complications were recorded in our series. Postoperative complications occurred in 37.5% of patients. All complications were in the early postoperative period (at least 3 months after surgery): one patient had urinary tract infection 12 days after surgery, and the other two had superficial wound dehiscence, both at one month after surgery. Urinary tract infection was treated with hydration and oral antibiotic therapy.

The two cases of superficial wound dehiscence were treated with wound surgical debridement and specific antibiotic therapy. In all cases, the complications were resolved. Only one patient died 11 months after surgery because of oncologic disease progression.

## 4. Discussion

Advances in 3D printing technology revolutionized the surgical approach to large bone defects in musculoskeletal oncology and revision surgery; this technology allows reconstruction of wide areas after bone resection tailored to the patient, as an alternative to large allograft or modular prostheses, ensuring a better anatomical fit with much more stability and strength in body weight bearing, due to biomechanical properties of porous titanium [12,15,16,25,26,27,28,29,30]. For example, for specific skeletal segments, such as scapula, clavicle and pelvic bones, there are not any modular prostheses available and reconstruction with massive bone grafting is associated with several disadvantages, including the risk of mismatch, immune rejection, fracture and infection [25]. Reconstruction with 3D customized prostheses is more accurate and stable, especially in these cases, with a rapid functional recovery.

Our study evaluates the application of 3D printed custom-made prosthesis in different sites. Angelini et al. [12] also analyzed 13 patients treated with 3D-printed prostheses in different oncologic and non-oncologic settings; they utilized three different implants and the surgeries were performed by different surgeons.

All patients in our study were treated by the same surgeon, an expert in oncologic musculoskeletal surgery, with a standardized protocol and with prostheses made by the same manufacturing company; moreover, all implants were silver coated. Silver’s antibacterial activity has long been recognized, and it has been used in the production of several biomedical devices, due to its low toxicity to human cells. Silver coating seems to be crucial in decreasing periprosthetic infection mostly in early postoperative period, due to its antimicrobial and also antifungal properties [30,31,32].

Oncologic patients are frequently weakened by the tumor itself and pharmacological treatments, because of this major invasive surgery and large implants surface could lead to an infectious complications increased risk [33].

The infection rate in oncologic orthopedic surgery ranges from 3.7% to 19.9% and increases up to 47% after pelvic resection and reconstruction [34].

As confirmed by many authors, silver coating reduces the re-infection rate on prosthesis, because of the releasing of silver ions, which produces an inhibition area, that makes the coated prostheses more resistant to infection in vivo [31].

Two patients developed early complications, consisting in superficial wound infection, treated with wound surgical debridement and antibiotic therapy, and in one case, a muscular flap was also needed. Those data are comparable to other studies in literature, even though in our work periprosthetic infection did not occur in any of the patients. Angelini et al. described in their study a complication rate of 38.5%, with four cases of wound dehiscence, two of whom then developed a deep infection [12]. Sun et al. found wound healing complications in 5 of 16 patients of their study, in one of them, prosthesis removal was needed because of deep wound infection [28].

We examined preoperative NLR and PLR, indicators of systemic inflammatory response that have recently been related to development and progression of several malignancies. It has been demonstrated that high blood levels of NLR and PLR in bone sarcomas could be suggestive of poor overall survival and disease-free survival [35,36].

Because of the limited number of patients in our study, it was not possible to relate the complications to those values; however, we observed that while NLR values were low (mean value 1.89) in all patients enrolled in our study, we found that PLR value was higher (177.78) in the patient who died. 

Mean intraoperative blood loss was 3.6 g/dL of hemoglobin; none of the patients required blood transfusion in the first postoperative day despite the considerable bone resection.

The resection margins were tumor-free and there were no local recurrences in any of our patients during the follow up.

The prostheses were employed to treat different massive bone defects in various areas and accurate soft tissue reconstruction and fixation over the prostheses, except for iliac bone implants, were achieved using Trevira Tube (Mutars^®^, Implantcast Corp., Buxtehude, Germany). Attachment tubes allow for a stable muscle connection on the entire prosthesis, giving a quicker and larger adhesion of muscles and tendons to the implant; this contributes to an additional mechanical resistance and stability and a more rapid recovery in musculoskeletal function after such demolition surgery [37].

We experienced positive functional outcomes with a high rate of satisfaction among our patients. Different scores were used to assess preoperative and postoperative quality of life among patients, evaluating different areas of patients’ life, such as physical performance, psychological aspects and the activities of daily living. We found that preoperative and postoperative quality of life were comparable. The patients preserved their autonomy on daily life activities with no functional impairment and they were able to carry out normal activities and work. Over 71% of the patients declared to be satisfied with the perception of their quality of life, giving a score that ranges from 8 to 10. Several studies in the literature evaluate functional outcomes using the Musculoskeletal Tumor Society Score, showing good results in terms of functional independence, pain relief, emotional acceptance and gait [6,8,11,12,14,28,29].

### Limitations

There are some limitations in our study, consisting principally of the small number of patients included. Liang et al. study [14] described the largest series, including 35 patients who underwent resection of a pelvic tumor and reconstruction using 3D printed endoprostheses.

Moreover, the procedures were all performed by a single experienced surgeon, the implant sites were multiple and different from each other and the follow up, although it consists of almost two years, is not long enough to show long-term outcomes. Furthermore, the analysis is retrospective and a selection bias may be possible.

## 5. Conclusions

Innovation in 3D printing technology has led to a new perspective in limb salvage surgery. Custom-made prosthesis permits reconstructing bone defects caused by large tumor resection, especially in anatomically complex areas, restoring articular function; this is a quickly developing field that, according to the results shown in literature, has encouraging outcomes in musculoskeletal oncologic reconstructive surgery. Additional studies with a larger population and follow up are needed to study the reliability and the efficiency of this kind of surgery.

## Figures and Tables

**Table 1:** Diagnosis and complications.

N Patient	Site	Primary Diagnosis	Complications	Complications Timing (Months after Surgery)
1	distal tibiae	metastasis	urinary infection	1
2	hip	sarcoma	wound dehiscence	1
3	total tibiae	adamantinoma		
4	iliac bone	chondrosarcoma		
5	distal femur	osteosarcoma	wound dehiscence	1
6	iliac bone	osteofibrous dysplasia		
7	proximal humerus	chondrosarcoma		
8	distal tibiae	sarcoma		

**Table 2:** Demographic data of our patients.

N pt	Gender	Age	N of Comorbidities	BMI
1	F	67	3	28.28
2	M	16	1	18.42
3	F	78	2	33.2
4	F	45	1	26.00
5	F	40	1	25.34
6	F	24	1	23.15
7	F	80	0	29.29
8	M	53	1	27.77
Mean		50.38		26.43
Standard Deviation		23.72		4.40

**Table 3:** Pre- and postoperative blood values. SD: Standard Deviation.

N pt	Hb Pre-Op	1st Post Op Hb	Pre Op Creatinine	Pre Op Albumin	Pre Op Neutrophils	Pre Op Linfocites	NLR	PLR
1	10.5	8.10	1.52	41	3.91	1.41	2.77	136.17
2	12.1	8.30	0.64	43	2.75	1.53	1.79	177.78
3	15.1	11.10	1.36	44	4.32	1.87	2.31	77.01
4	16.2	9.40	0.96	44	2.98	2.47	1.22	72.47
5	12.5	11.70	1.02	42	4.08	1.63	2.50	107.36
6	12.9	8.00	0.77	39	4.19	2.09	2.00	120.09
7	13.6	10.90	0.92	42	4.30	2.32	1.85	43.95
8	13.0	9.10	0.67	37	4.72	3.08	1.53	56.49
Mean	13.24	9.58	0.98	41.50	3.91	2.05	2.00	98.92
SD	1.77	1.47	0.32	2.45	0.69	0.56	0.51	44.92

**Table 4:** Patients’ outcomes. (ADL: activities of daily living; IADL instrumental activities of daily living; SF 12M: SF12 mental; SF 12 P: SF12 Physical; SD: Standard Deviation).

	Pre Op ADL	Pre Op IADL	Post Op ADL	Post Op IADL	Delta ADL	Delta IADL	Pre Op Karnofsky	Post Op Karnofsky	Pre Op SF 12 M	Pre Op SF 12 P	Post Op SF 12 M	Post Op SF 12 P	Delta SF12 M	Delta SF 12 P
1 PA	//	//	//	//			//	//	//	//	//	//	//	//
2 SAI	4	4	4	4	0	0	70	70	12	13	12	13	0	0
3 MF	4	4	4	4	0	0	70	70	11	14	11	14	0	0
4 FFM	6	6	6	6	0	0	90	90	11	15	11	15	0	0
5 CL	4	4	5	5	1	1	80	80	12	14	13	13	1	−1
6 SB	6	6	5	5	−1	−1	80	80	12	13	11	14	−1	1
7 FP	4	4	5	5	1	1	80	80	12	15	11	16	−1	1
8 RV	6	6	6	6	0	0	100	100	12	16	12	16	0	0
Mean	4.86	4.86	5.00	5.0	0.13	0.13	81.43	81.43	11.71	14.29	11.57	14.43	−0.14	0.4
SD	1.07	1.07	0.82	0.82	0.64	0.64	10.69	10.69	0.49	1.11	0.79	1.27	0.69	0.69

## Data Availability

The datasets used and/or analysed during the current study are available from the corresponding author on reasonable request.

## Abbreviations

Activity Daily Living: ADL; Instrumental Activity Daily Living: IADL; Short Form-12: SF-12; Platelet/lymphocyte ratio: PLR; Neutrophil/Lymphocyte ratio: NLR; Magnetic resonance imaging: MRI; Karnofsky performance status: KPS.
